# Comparison of Accuracy in Expert Clinical Examination versus Magnetic Resonance Imaging and Arthroscopic Exam in Diagnosis of Meniscal Tear

**DOI:** 10.1155/2020/1895852

**Published:** 2020-05-08

**Authors:** Seyed Ali Hashemi, Mohammad Reza Ranjbar, Mohammad Tahami, Reza Shahriarirad, Amirhossein Erfani

**Affiliations:** ^1^Research Center for Bone and Joint Diseases, Department of Orthopedic Surgery, Chamran Hospital, Shiraz University of Medical Sciences, Shiraz, Iran; ^2^Student Research Committee, Shiraz University of Medical Sciences, Shiraz, Iran; ^3^Thoracic and Vascular Surgery Research Center, Shiraz University of Medical Sciences, Shiraz, Iran

## Abstract

**Background:**

Many clinical tests and diagnostic studies have been developed to increase the clinician's ability to accurately diagnose disorders of the knee. Torn menisci or ligamentous structures within the knee cause significant pain and disability and thus require expeditious management. This study was conducted to evaluate the accuracy of clinical examination in comparison with MRI examination and with the help of arthroscopic examination as the gold standard in the diagnosis of meniscal tears.

**Method:**

All of the arthroscopic surgery candidates, presenting symptoms of meniscal or cruciate ligament lesions, referring to Namazi and Chamran hospitals, Shiraz, Iran, were included in this study. Clinical examination (including McMurray test, Apley test, and 20 Thessaly test) was performed before the arthroscopy, and the results were recorded in special forms. Magnetic resonance imaging (MRI) results were also added. Then, arthroscopy was performed, declaring the definite diagnosis, and the results were compared to the results obtained from both tests and MRI. Statistical analysis was performed using SPSS software.

**Results:**

86 patients with a mean age of 27 years old, including 63 (73%) male and 23 (27%) female, were studied. 57 (66%), 19 (22%), and 10 (12%) injuries were caused by sports, twisting, or trauma, respectively. Arthroscopic results showed 32 meniscal tears, of which 28 (87%) and 4 (13%) were in medial and lateral menisci, respectively, including 10 bucket handle, 17 longitudinal, and 5 of other types (transverse, oblique, radial) of injuries. Comparing MRI results to arthroscopic results, we had 2 false-positive and 2 false-negative cases. 62 cases of McMurray test results were accurate; 15 and 9 cases were reported false positive and false negative, respectively. 60 cases of Apley test results were accurate; 16 and 10 cases were reported false positive and false negative, respectively. 78 cases of Thessaly test results were accurate; 5 and 3 cases were reported false positive and false negative, respectively. Comparing Thessaly test results to McMurray and Apley showed statistical significance (*P* < 0.05). Comparing Thessaly test results to MRI showed no statistical significance (*P* = 0.151), while comparing McMurray and Apley test results to MRI showed statistical significance (*P* < 0.01).

**Conclusion:**

Clinical examination, performed by an experienced examiner, can have equal or even more diagnostic accuracy compared to MRI to evaluate meniscal lesions. In this study, the Thessaly test has been approved as a reliable clinical test in the diagnosis of meniscal tears.

## 1. Introduction

Diagnosis of acute knee injuries has long been one of the issues discussed in orthopaedic sources. Many clinical trials and diagnostic studies have been conducted to increase the ability of physicians to correctly diagnose knee problems [1–3]. Since ruptured meniscus or ligamentous structures in the knee cause significant pain and disability, these lesions require prompt and accurate treatment and management. Due to the devastating consequences of meniscus injuries in patients, especially for those injured during exercise, timely and accurate diagnosis of these injuries is essential [4].

The necessity of surgery after meniscus injury is significantly determined by initial physical examination along with other diagnostic tests. A comprehensive examination should include a full description of the injury, palpation of the injury site, and a selection of specific tests. Athletes with meniscus tears usually describe a pop-like sound when redirecting in a sprint, such as twisting the heel of the foot with or without colliding with another player [5].

Joint line tenderness and effusion are also manifestations of meniscal injury. Palpation of the knee along the axis may have indefinite results in predicting meniscus ruptures with an acute ACL injury, with a specificity of 34.5% and sensitivity of 44.9% based on internal axis palpation and a specificity of 49.1% and sensitivity of 57.6% for external axis palpation. In cases where the ACL is unaffected, tenderness along the joint line more accurately (77%) identifies meniscal ruptures [5].

The main tests required to treat patients with symptoms so far include palpation of the joint border, McMurray test, Apley grind test, and Thessaly test [6, 7].

Arthroscopy is the gold standard of diagnosis in traumatic injuries to the knee joint. Arthroscopy, although highly accurate, is an invasive and expensive intervention that requires hospitalization and general or regional anesthesia, but it can also impose complications on an open surgery, such as infections, neurological and vascular injuries, and injury to the intra-articular elements of the knee [8, 9]. With 1,200 knee arthroscopies a year and rising, a noninvasive diagnostic tool that requires no intra-articular approach could minimize the potential risks of arthroscopy and reduce the numbers of needed arthroscopies [10]. Although the diagnostic significance of a physical examination by an experienced clinician has been evaluated in various studies [10, 11], further studies are justified for whether or not it could be favored over other diagnostic methods. Therefore, this study was conducted to evaluate the accuracy of clinical examination in comparison with MRI examination and with the arthroscopic examination as the gold standard in the diagnosis of meniscal tears.

## 2. Materials and Methods

This cross-sectional descriptive study was performed on all patients who referred to Shahid Chamran and Namazi hospitals of Shiraz with any signs of trauma to the meniscus or cruciate ligament, whether due to accidents or athletic trauma, and who were candidates for arthroscopic surgery. The study protocol was approved by the Ethics Committee of the Shiraz University of Medical Sciences.

Participants were selected by available sampling from patients who referred to Shahid Chamran and Namazi hospitals. Patients with trauma due to accidents or athletic trauma who were candidates for meniscus or cruciate ligament arthroscopic surgery were enrolled in our study. Patients who had positive examination in favor of meniscus injury and who had a clinical complaint were treated with arthroscopy, regardless of the MRI result. Also, patients who had imaging results in favor of meniscus injury with normal physical examination who underwent arthroscopy were included in the study. Eventually, 86 patients were enrolled in our study. All cases that had a history of knee surgery, knee arthroscopy, repair of ligaments or meniscus, or history of meniscectomy, either in total or partial, were excluded from the study.

Examinations were performed with competence by an experienced orthopaedic surgeon, with no knowledge of the patients' MRI results, before performing arthroscopy. All performed clinical examinations and the patient characteristics were entered in prepared specific forms for this purpose. Eventually, MRI results were added. The tests used in this study included the McMurray test, the Apley test, and the 20-degree Thessaly test. Then, the MRI images of each patient were examined by a qualified diagnostic radiologist expert and their results were reported. Finally, arthroscopy was performed by an experienced and qualified orthopaedic surgeon and with the help of his assistants; results were recorded as a definitive diagnosis; comparing the results with the results obtained from the tests and MRI, true positives and negatives and false positives and false negatives were obtained.

Statistical analysis was performed using SPSS (version 18; SPSS Inc., Chicago, IL, USA) and McNemar and chi-square (*X*^2^) tests. The findings of this study were evaluated using descriptive statistics and sensitivity, specificity, accuracy, positive predictive value (PPV), and negative predictive value (NPV). The chi-square and McNemar tests, each based on their use, were used to compare the findings of the tests and MRI. Finally, the results of the comparison with *P* value less than 0.05 were considered statistically significant.

## 3. Results

In this study, 86 patients were enrolled with a mean age of 27 years (range: 15 to 45 years).  Table 1 demonstrates the frequency and location, along with the types of injury findings by arthroscopy in our study.

Arthroscopic results revealed 32 cases of meniscus rupture, of which 28 (87%) were medial meniscus injuries and 4 (13%) were lateral meniscus injuries. Of these, 23 (72%) were male and 9 (28%) were female, with 13 (41%) cases of left knee injuries and 19 (59%) cases of right knee injury. Arthroscopy also showed a wide range of injuries including 10 bucket handle injuries, 17 longitudinal injuries, and 5 other types including transverse, oblique, and radiated or radial injuries.

Concerning the MRI imaging protocols and comparing them with the results of arthroscopy, the accurate results included 82 cases of which 30 (34.88%) were positive and 52 (60.48%) were negative. Among the 4 reported error cases, we had 2 false positives (2.32%) and 2 false negatives (2.32%). Of the two false positives, one had a longitudinal rupture and the other radial rupture; among the two false positives, one was a longitudinal rupture and the other was bucket handle.

Using descriptive statistics, the sensitivity, specificity, and diagnostic accuracy of MRI were calculated to be 93.7 (79.2–99.1), 96.3 (2–99.4), and 95.35%, respectively.

Among the 86 cases examined, the results were as follows.

62 of all McMurray test cases were accurate. Of the 24 errors, 15 were false positive, of which 3 were in the lateral meniscus and 12 in the medial meniscus, and 9 cases, including 1 in the lateral meniscus and 8 in the medial meniscus, were reported false negative.

Sixty out of all Apley test cases were accurate. Of the 26 errors, 16 were false positive, 2 of which were in the lateral meniscus and 14 in the medial meniscus, and 10, including 1 in the lateral meniscus and 9 in the medial meniscus, were reported false negative.

78 of all Thessaly test cases were correct. Of the 8 errors, 5 were false positive, 1 in the lateral meniscus and 4 in the medial meniscus. However, among the false positives, one was discoid meniscus and another involving articular cartilage injury. Also, 3 cases, including 0 in the lateral meniscus and 3 in the medial meniscus, were reported false negative.  Table 2 demonstrates the frequency of rupture types in false-negative and false-positive examinations.

Using descriptive statistics methods for McMurray, Apley, and Thessaly tests, sensitivity, specificity, accuracy, positive predictive value (PPV), and negative predictive value (NPV) were reported, as shown in Table 3.

## 4. Discussion

This study showed that the diagnostic power of experts' physical examination and MRI was high in meniscus injuries, although there were some differences. For example, the diagnostic power of MRI was higher than that of the tests performed on physical examination (McMurray, Apley, and Thessaly tests), as the results also showed greater sensitivity, specificity, and diagnostic accuracy of MRI than these tests, and among these tests, the diagnostic power of the Thessaly test was higher than that of the other two tests, as presented by higher sensitivity, specificity, accuracy, and positive and negative predictive values which were reasons to support this claim.

The results of our study in this area are in contrast to the results of a study by Navali et al. [12], which also emphasizes the high diagnostic power of experts' physical examination and MRI in meniscus injuries, but point to the importance of physical examination over MRI in their study, which is emphasized in the results of their study and also supported by other studies [7, 13–16].

Contrary to these studies, a study by Nikolaou et al. [17] acknowledges less diagnostic power of physical examination than MRI, which is consistent with the results of the present study. However, studies have shown that if a physical examination is performed by well-skilled and qualified personnel, it can produce results with accuracy similar to MRI, which can then be used as an appropriate diagnostic tool [12].

In general, the results of physical examinations for the diagnosis of lateral meniscus lesions were superior to those of medial meniscus. However, Navali's study points to the superiority of physical examination in the diagnosis of medial meniscus injuries [12].

In a meta-analysis by Wang et al., the international specificity and sensitivity of MRI of meniscal tears were 95.0% (95% confidence interval (CI): 91.0–97.0%) and 80.0% (95% CI: 66.0–89.0%) in lateral meniscal tears and 90.0% (95% CI: 85.0–95.0%) and 92.0% (95% CI: 88.0–95.0%), respectively [18].

In a study by Yan et al. [19], as in our study, MRI was introduced as a sensitive method for diagnosis of meniscus rupture. Although in a study by Ben-Galim et al. [20], false positives reported in MRI diagnosis were reported 65% for medial meniscal rupture and 43% for lateral meniscal rupture but has been recommended alongside physical examination due to high diagnostic accuracy and high negative predictive value. However, in our study, false-positive MRI cases accounted for only 2.32% of the cases, which confirms this method due to its high diagnostic accuracy (95.35%).

The surgeon can decide, solely relying on the clinical examination, whether further examination via MRI is necessary or whether the patient can be admitted for surgery. Lack of practice and experience in the examiners leads to variable accuracy in this decision. Bohnsack et al. [21] established that an experienced examiner can diagnose effectively by only clinical examination, in which in their study a clinical examination executed by an experienced surgeon was 80% precise for diagnosing lateral meniscal tears and 93% accurate for detecting medial meniscal tears while for the least experienced examiner in their study, which was a fourth-year resident, the clinical examination was 80% accurate for diagnosing lateral meniscal tears and 73% accurate for identifying medial meniscal tears. This linked to an MRI accuracy of 83% for both lateral and medial meniscal tears. Therefore, their results showed that a clinical examination performed by an experienced surgeon is more valuable than an MRI scan in the diagnosis of meniscal injuries [7].

In a study by Karachalios et al. [22], they reported a 20-degree Thessaly test with diagnostic accuracy of 94% in the diagnosis of medial meniscal rupture and 96% accuracy in the diagnosis of lateral meniscal rupture as well as small false positive and negative values, introducing the test as the first-line in clinical screening for meniscal rupture and has been recommended as a suitable alternative to costly MRI.

The results of comparing the Thessaly test with each of the McMurray and Apley tests were also statistically significant (*P* < 0.001), indicating the superiority of the Thessaly test over others. As also shown in our study, the rest of the indices (sensitivity of 90.6%, specificity of 90.7%, accuracy of 90.69%, and positive and negative predictive values 85.3 and 94.2) were also higher in this test compared to the two other tests. In a study by Harrison et al. [23] on 116 patients (with 66 Thessaly positive test cases), they reported a 90.3% sensitivity, 97.7% specificity, and 88.8% diagnostic accuracy for the Thessaly test and has been introduced as a valid test in the detection of meniscal rupture, which can replace older tests. As can be seen, the results of this study are consistent with our study. Also, the McMurray and Apley tests were compared in our study, with no statistically significant difference (*P*=0.267). It should be noted that the error rates of these two tests were almost similar, nevertheless, considerably higher than the Thessaly test. The Karachalios study also highlights false positives in tests such as McMurray [22].

## 5. Conclusion

Many clinical trials and diagnostic studies have been conducted to increase the ability of physicians to correctly diagnose meniscus injuries. One noninvasive alternative to arthroscopy to diagnose meniscus injuries is MRI, which is used today to diagnose meniscus injuries before performing arthroscopic and surgical examinations. The present study showed that MRI with high sensitivity and specificity and significant diagnostic power could be useful in the diagnosis of meniscal ruptures and knee ligaments.

Performing a rigorous physical examination by an experienced examiner has the diagnostic power equal to or better than MRI in the determination of meniscal lesions. In this review, it was found that the Thessaly test can also be used as an appropriate physical examination to detect meniscal rupture in comparison with other tests.

## Figures and Tables

**Table 1:** Frequency and location, along with the types of injury findings by arthroscopy in patients with meniscus injury.

	Frequency	Percent
*Age*		
15 to 25 years	22	25.58
25 to 35 years	45	52.32
35 to 45 years	19	22.10

*Gender*		
Male	63	73
Female	23	27

*Cause of injury*		
Exercise	57	66
Trauma/torsion	19	22
Accidental injuries	10	12

*Location*		
Right knee	53	61.6
Left knee	33	38.4

*Arthroscopy injury*		
Medial meniscus injury	28	87
Lateral meniscus injury	4	13
Bucket handle tear	10	31.25
Longitudinal tear	17	53.12
Transverse/oblique/radial tear	5	15.63

**Table 2:** Frequency of rupture types in false-negative and false-positive examinations.

	False negative			False positive	
	Type of injury			Medial meniscus	Lateral meniscus
	Bucket handle	Longitudinal	Transverse/oblique/radial		
Thessaly	1	1	1	4	1
McMurray	2	5	2	12	3
Apley	2	6	2	14	2

**Table 3:** Sensitivity, specificity, accuracy, positive predictive value, and negative predictive value in Thessaly, McMurray, and Apley tests.

Type of test	Accuracy (%)	Negative predictive value (%)	Positive predictive value (%)	Specificity (%)	Sensitivity (%)
Thessaly	90.69	94.2	85.3	90.7 (79.7–96.9)	90.6 (75.0–97.9)
McMurray	72.09	81.3	60.5	72.2 (58.5–83.5)	71.9 (53.3–86.2)
Apley	69.77	79.2	57.9	70.4 (59.4–82)	68.7 (50–83.9)

The results of the comparison of the Thessaly test with each of the McMurray and Apley tests were statistically significant (*P* < 0.001); also, the McMurray and Apley tests were compared with each other with no significant difference (*P*=0.267). Findings from each of the Thessaly, McMurray, and Apley tests were also compared with the MRI results, which unlike the McMurray and Apley tests, were not statistically significant for the Thessaly test (*P*=0.151).

## Data Availability

SPSS data of the participants are available from the corresponding author upon request.
